# Collective Irrationality and Positive Feedback

**DOI:** 10.1371/journal.pone.0018901

**Published:** 2011-04-26

**Authors:** Stamatios C. Nicolis, Natalia Zabzina, Tanya Latty, David J. T. Sumpter

**Affiliations:** 1 Mathematics Department, Uppsala University, Uppsala, Sweden; 2 School of Biological Sciences, University of Sydney, Sydney, New South Wales, Australia; University of Zaragoza, Spain

## Abstract

Recent experiments on ants and slime moulds have assessed the degree to which they make rational decisions when presented with a number of alternative food sources or shelter. Ants and slime moulds are just two examples of a wide range of species and biological processes that use positive feedback mechanisms to reach decisions. Here we use a generic, experimentally validated model of positive feedback between group members to show that the probability of taking the best of 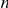 options depends crucially on the strength of feedback. We show how the probability of choosing the best option can be maximized by applying an optimal feedback strength. Importantly, this optimal value depends on the number of options, so that when we change the number of options the preference of the group changes, producing apparent “irrationalities”. We thus reinterpret the idea that collectives show "rational" or "irrational" preferences as being a necessary consequence of the use of positive feedback. We argue that positive feedback is a heuristic which often produces fast and accurate group decision-making, but is always susceptible to apparent irrationality when studied under particular experimental conditions.

## Introduction

Several recent studies have begun to investigate the collective rationality of distributed biological systems [1], [2]. A striking result is that acellular slime mould *Physarum polycephalum* makes irrational decisions, in the sense that its preference for food items of varying quality changes when its choice set is expanded [1]. These results are reminiscent of choice patterns seen in humans participating in decision-making tasks, where the relative attractiveness of two options often depends on the presence or absence of a third option [3], [4]. Such preference changes violate independence from irrelevant alternatives (IIA), because a new option of lesser value apparently alters the value of the two superior options, and can thus be classified as ‘irrational’ [5]. In addition to slime moulds, rationality has been studied in house-hunting *Temnothorax* ants. Unlike slime moulds, *Temnothorax* ants are, in some situations, collectively immune to irrationality and do not violate IIA [2], [6].

Several authors have pointed out that rationality cannot be studied in isolation from mechanisms [4], [7]. Gigerenzer emphasises the importance of heuristics, which are fast methods for making decisions on the basis of only small amounts of available information [8], [9]. The key questions to ask in such a framework are “In what environments will a given heuristic work? Where will it fail?” [9]. For collective decision-making, the key heuristic is positive feedback, whereby commitment to a particular option increases as a function of the number of individuals already committed to it [10]–[12]. In the light of the new experiments classifying ants and slime mould as “rational” or “irrational”, it is important to link these outcomes to the positive feedback heuristic: in what environments do we expect positive feedback to produce accurate decisions and in what circumstances do we expect it to fail?

For *Physarum* and *Temnothorax* ants, the feedback mechanisms by which these systems reach decisions are relatively well understood. For *Physarum*, positive feedback is mediated through the growth of tubes as a result of protoplasmic flow [13]. Positive feedback in *Temnothorax* ants is in the form of tandem running which recruits nestmates to good quality nests with a switch to a rapid transport of nestmates after a quorum threshold is reached [14]. In this paper, we consider the problem of choosing between multiple options in a general model of positive feedback supported by experimental evidence, namely the Deneubourg model of collective decision-making [15]. The model describes two or more competing positive feedback loops, each of which measures how the build up of commitment to a particular option evolves in time.

We assume that each option has an associated quality encoded by the variable 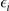. We also assume that commitment decays at a constant rate 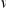 for each option. The evolution of the commitments 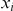 to option 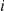 can thus be cast in the form [10]

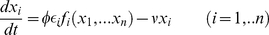
(1)where the flux 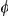 determines the overall strength of positive feedback. Note that for animal groups 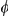 can be thought of the number of individuals per time step making a decision, or as proportional to the size of the population.

The choice function 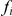 (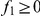) expresses how future commitment to 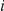 is affected by the current commitment both to 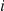 and its competitors 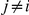. 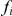 is chosen to provide a quorum-like response, so that above a threshold the rate of increase in commitment becomes significantly larger. The detailed mechanisms behind the resulting positive feedback and the specific forms of 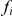 depend on the system at hand. In *Physarum* 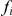 is a nonlinear saturating function modeling the increase in flow as a function of tube thickness [16]. Other mechanisms generating quorum-like positive feedbacks are trails formation [10], [17], following behavior by fish [18], interactions between individuals in connection with aggregation like processes [19]–[21] and imitation by primates [22]. Imitation is also the dominant feedback mechanism in the context of decision-making phenomena involving human populations. The situation for *Temnothorax* ants is more complicated. For these ants 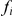 can be considered linear, since tandem running recruits ants in proportion to the number of ants already recruiting, but there is a quorum switch to transporting of nestmates above a certain threshold [14]. In what follows we will use a form of 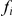, inspired by theoretical and experimental work on food recruitment in social insects by Deneubourg and co-workers [17], [23], [24], written as
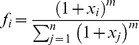
(2)


The parameter 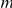 measures the sensitivity of the particular choice. The larger the 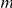 the sharper the choice and, at the same time, the higher the nonlinearity involved in the process. In this paper, we will study the reference case where 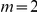 corresponding to the minimal setting of co-operativity, and subsequently see if the conclusions persist for different values of this parameter.

## Results and Discussion

**Figure 1:**
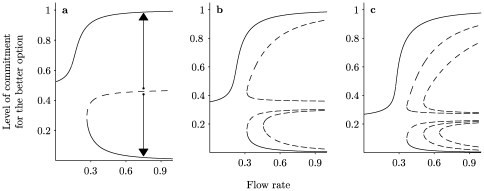
Bifurcation diagrams of 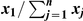 corresponding to the steady state level of commitment for the better option (eqs. (3) – (6))with respect to the flow rate 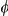. (a) case 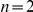, (b) 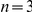 and (c) 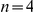. Full and dashed lines correspond to stable and unstable solutions respectively. The stability has been checked numerically by integrating the full eqs. (1) – (2). The arrows indicate the evolution of initial conditions on the two sides of a threshold value corresponding to the intermediate unstable state. Parameter values are 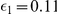, 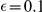, 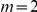 and 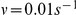.

Figure 1 shows the bifurcation diagrams of the steady state solution of eqs.(1)–(2), i.e., how the steady state level of commitment for the best option changes with the flux 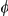 for 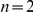, 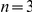 and 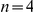. As the flux increases the system switches from having one stable state with a small majority committed to the option with the highest value of 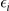 to having multiple steady states with stronger commitment to one of the options. In this latter situation, there is one steady state corresponding to a high level of commitment to the highest quality option, but there also exist alternative stable states corresponding to commitment to one or more of the lower quality options. Here, the chosen option depends on initial conditions. For example, in figure 1a the arrows show how different initial commitment levels will evolve. If commitment is initially strong for the lower quality option then the system moves towards choosing this option. We do not label such a situation as ‘irrational’ since it is common to see humans and other animals having a range of possible choices depending on their initial preferences. Indeed, it may well be optimal to choose the option closer to an initial preference.

We can however show that it is this multi-stability that can lead to irrationality when the number of choices available to a decision-maker is changed. The first point to note is how the bifurcation diagrams in figure 1 depend on the number of options. In particular, the bifurcation point where more than one steady state appears increases with the number of options. For 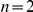 this bifurcation point is 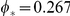, for 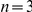 it is 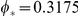, and for 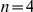 it is 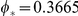. We note also that the stable branch corresponding to a majority commitment to the higher quality option moves to the right as the number of choices increases. In other words, the preferences change with the number of options.

The quality of a decision does not simply depend on whether or not the best option is chosen by more individuals than any of the other options. The size of the level of commitment is also important. For example, in figure 1b when 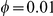 only a very small 36% choose the best option, while 32% choose each of the two poorer options. This is to be compared to 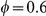 where 96% choose the best option. Thus the quality of decision can be defined as a combination of (a) the proportion of individuals committed to the better option and (b) the proportion of cases where this option is selected over the less favorable one. This latter quantity depends on initial conditions and/or random factors and cannot be calculated from eqs.(1) – (2) alone. We therefore use our Monte Carlo simulation to calculate (b) for 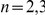 and 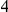 (see materials and methods).

**Figure 2:**
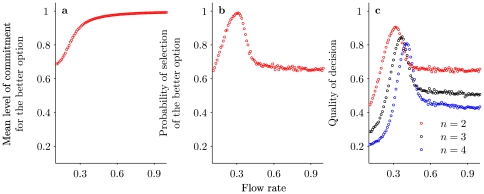
Quantitative view of the quality of decision as a function of the flow rate 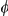 as obtained by Monte Carlo simulations. (a) Mean level of commitment for the better option 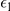, (b) Probability of selection of the better option and (c) Quality of decision for three different numbers of options. Parameter values as in Fig. 1, number of realizations is 5000.

Figure 2a presents the proportion of individuals 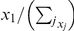 selecting the higher quality option, averaged over many Monte Carlo realizations. This plot corresponds to the upper branch of Figure 1a. Figure 2b provides the number of cases in which the higher quality option is preferred over the total number of Monte Carlo realizations (i.e., cases in which 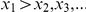 at steady state). Figure 2c multiplies these curves pointwise to provide an overall measure of how quality of decision depends on flow rate 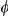 for different numbers of options. In all cases, the strongest bias to the highest quality option occurs near to the bifurcation point, 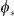, where the system goes from one to more than one steady state. This result can be contrasted to Condorcet's theorem, which states that large groups are able to make better decisions [25]–[27]. More recently the effect of the presence of an expert has also been considered [28]. Here we have shown that the quality of decision is not just a matter of population size (in our model captured by the flow parameter 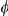) but is affected by the strength of the positive feedbacks along with the number of options presented.

Decision-making is often associated with a speed-accuracy trade-off, where a more accurate decision requires more time to reach [12], [29], [30]. Recent theoretical and experimental work has shown that such a tradeoff does not always hold. For example, Katsikopoulos et al. [7] show using computer simulations that a ‘take the best’ heuristic method can make better decisions using less computational effort without suffering an accuracy loss. Predator avoidance experiments on fish have shown that larger groups make decisions more rapidly and more accurately than smaller groups or solitary fish [31]. To address this question in our system, we consider the rate at which a decision is reached as we increase the flow parameter 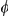. This rate is determined by the largest eigenvalue, which we will denote 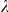, for the steady state corresponding to the better option. The time taken to reach a decision is proportional to the inverse of the magnitude of 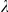 (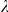 is always negative). As we increase 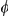, the magnitude of 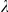 increases and thus the time to reach a decision decreases. Conversely, reducing the flow parameter to a level below 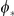 (i.e. the point at which accuracy is maximized) will lead to a slower, as well as a less accurate decision. In short, there is no trade-off between speed and accuracy here, rather there is an optimal parameter where both are maximised.

**Figure 3:**
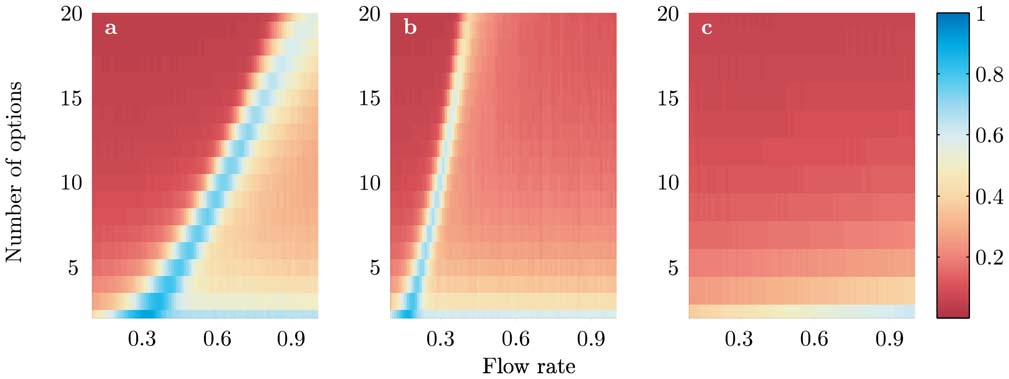
Decision quality as a function of the flow rate of individuals 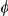 and of the number of options 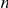 as obtained by Monte Carlo simulations. (a) case 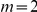, (b) 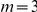 and (c) 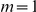. Other parameter values as in Fig. 1, number of realizations is 5000.

Our results hold for any number of options. Figure 3a gives this quality measure for a complete parameter scan of the model for multiple options and a full range of flows 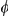. Again, the maximal decision quality occurs near the bifurcation point, 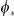. The position of the bifurcation point increases linearly with the number of options. For 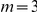 (see Figure 3b), the situation resembles to our canonical case 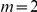 except that the transition from one to more than one steady state is sharper, which is not very surprising as 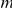 can be viewed as a parameter controlling the accuracy of the decision. Our results appear robust provided 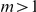. This is exactly the conditions for a quorum-like response in the positive feedback. The exception, where there is no quorum-like response, is 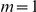 (figure 3c) and we discuss this case further below.

**Figure 4:**
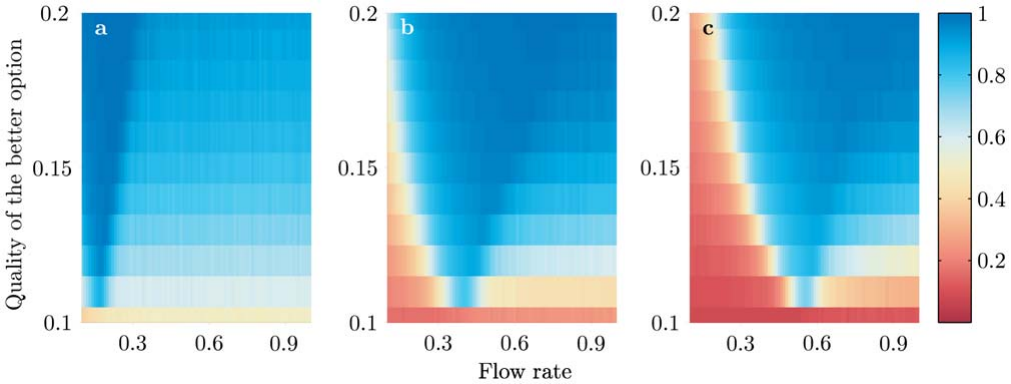
Decision quality as a function of the flow rate of individuals 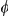 and of the quality of the better option 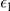 (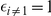) as obtained by Monte Carlo simulations. (a) case 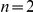, (b) 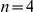 and (c) 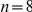. Other parameter values as in Fig. 1, number of realizations is 5000.

Until now, we assumed that the best option was slightly higher in its quality than the other one (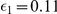, 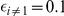). In Figure 4 we show the combined effect of an increasing option quality and an increasing flow rate on the quality of decision for three different numbers of options. The region where the quality of decision is near maximal becomes wider as 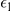 increases. On the other hand when the number of options increases the maximum is shifted towards higher values of the flow rate and of the quality of the better option.

For any given number of options the decision-making outcome is different. In particular, the flow level at which the highest quality option is chosen most often depends on this particular number. In a situation where it is optimal to pick the highest quality option irrespective of initial conditions, then we can see that a value of 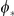 which is optimal for choosing between, for example, two options is not optimized for choosing between three or more options. Likewise, a flow level which might make good decisions between four options can perform poorly when faced with a decision between two. This result is highly counterintuitve, since we would not expect a system to make a worse decision when confronted with a smaller number of options. Indeed, it is here that we can talk about a system behaving irrationally. Alternative dummy choices adjust the outcome of choice experiments, and in such a way that an obviously better option is chosen less often.

Experiments testing the effect of additional choices in decision-making do not start from the premise that one option is always optimal, independent of initial conditions. For house hunting ants or foraging slime mould there is a cost to be paid in switching between options if there is already an established commitment for one particular option. Indeed, when offered two options of similar although not identical quality over multiple trials, ants and slime mould do not aggregate at the same option in every trial [1], [2]. Instead they choose the one which elicits the greatest initial build-up of commitment [16], [32]. Our model shows that introducing a third option in such situations will always change the expressed preferences. In figure 2 we introduce a third option of equal quality to the second, but the results are not contingent on these two options being the same. Rather, the introduction of additional options always has the potential to reveal ‘irrationality’ in experimental situations.

Species differences can, through our model, explain differences in the outcome of rationality experiments. Positive feedback is very strong in slime moulds, suggesting 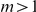. Tube connections between food sources build rapidly, the slime mould will usually choose only one of many identical food sources [16] and the tubes remain stable even when conditions change [33]. This may explain why the choice patterns of slime mould depend so strongly on the number of options presented. The situation is reversed for ants. Here tandem running provides a weak positive feedback and when the number of choices is increased the ants preferences remain stable [2]. Roughly speaking, weak feedback corresponds to setting 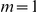, although see Pratt et al. [14], [34] for a more detailed model of *Temnotorax* emigration. Figure 3c shows that the parameter scan looks very different than 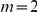. Here, there is only one solution to equations (1) – (2). This corresponds to the better option being selected 100% of the cases, but with a relatively low level of commitment. In this case, the proportion of commitment increases linearly with the flux for a particular number of options, and decreases again smoothly with the number of options for a particular value of the flux.

Many of the standard models of decision-making assume that choice is a linear process [8], [35]. For example, Busemeyer and Townsend use a linear stochastic difference equation for the change of the preference state in the course of time [4]. This equation (cf. eq. (7) of ref. [4]) features in turn quantities indicating the propensity to choose the different options which bear some resemblance with our choice functions 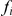, eq.(2), the main difference being the absence of cooperativity. In order to reproduce violations of independence of irrelevant alternatives, these models include updating rules in which comparison between options influence the strength by which various options are preferred. Here, we have shown that IIA violating outcomes can arise naturally from the underlying dynamics without varying comparison schemes, provided we have a non-linear choice function with 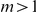. There is strong experimental evidence in amoeba and social insects that such non-linear feedbacks are present, and they may well be present in behavioral science and particularly in psychology as well. Indeed, in terms of our criteria (a) and (b) for the quality of decision-making (cf our earlier comments in connection with figure 2), 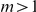 is superior to 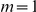 and could thus be expected to be a widely used heuristic.

Models of decision-making in the visual cortex and other areas of the brain usually assume feedbacks between groups of neurons, with each group accumulating evidence for a particular option [36], [37]. There are strong parallels between the positive feedback system described by Deneubourg's and other models of social insect decision-making and these neuronal models, although in the latter case the focus is usually on cross-inhibition [38]. As a consequence some functions of the human brain are likely to be subject to the same constraints resulting from the model presented here. In particular, if we change the number of options in a decision-making situation, we expect the corresponding stability of steady states to change and as a result choice preferences will also change. This is exactly what is observed in choice experiments on individual humans and animals [3], [39], [40]. So called "irrationality" could again be a here a consequence of the mechanisms employed within the brain. More work is certainly needed to test this idea. One of the interests of our approach has been to raise the issue of rationality in the context of cellular and population biology where quantitative experiments can be carried out in detail and modeling and simulation approaches calibrated by experimental data can be developed.

In summary, we have shown here that concepts such as increasing accuracy with group size, speed-accuracy tradeoffs and "irrational" decisions are strongly correlated to the coexistence of multiple stable steady states. In the context of systems based on positive feedback, "rationality" and "irrationality" appear in some respects to be terms for describing the possibility that decisions can have different outcomes dependent on initial conditions. In particular, "irrationality" can be created in such systems simply by conducting an experiment in which the positive feedback is sufficiently high to generate multiple steady states. The question is not then whether a system is "irrational" or not, but rather why it uses strong positive feedback?

## Materials and Methods

We study the above model in two ways, both as a system of differential equations as defined by eqs.(1) – (2) and as a Monte Carlo simulation. In the latter case, decision-making is modeled as a stochastic process of transitions towards states whose probabilities, given by 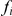 are being continuously updated as the process is advancing in time. More specifically, the simulation starts with the number of individuals on each option equal to zero. The first decision concerns the individuals to choose or not to choose, given by a probability equal to 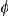. The second decision is the actual choice of one option, governed by eq.(2). During time evolution, when an individual chooses an option 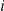, it reinforces the probability 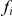 to choose in the future that option but at the same time there is a fixed rate at which individuals abandon the option (parameter 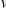). The process is repeated for a number of steps sufficient to reach the stationary state.

We focus on the situation in which one option is better than all the other ones considered to be of equal quality (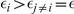). The mean field equations (1) – (2) yield then in the steady state explicit expressions for the 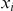
's. One has successively,
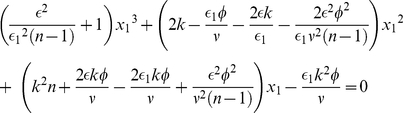
(3)with
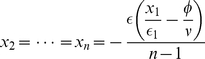
(4)and
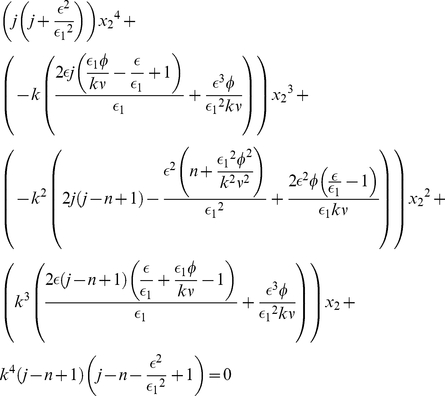
(5)with
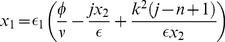
(6)being understood that in eqs.(5) – (6) the population splits into two groups choosing respectively 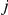 options such that 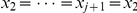 and 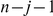 options such that 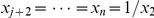, the better option 1 still being chosen by 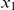.

A typical way to summarize the behavior of the solutions of eqs.(3) – (6) is to draw bifurcation diagrams as in Fig. 1 in which the value of the relevant variable at the steady state is plotted against one of the parameters present in the problem.
